# Assessing health literacy in the eastern and middle-eastern cultures

**DOI:** 10.1186/s12889-016-3488-9

**Published:** 2016-08-19

**Authors:** Satish Chandrasekhar Nair, Karthyayani Priya Satish, Jayadevan Sreedharan, Halah Ibrahim

**Affiliations:** 1 Academic Affairs Medical Affairs Department, Tawam Hospital- Johns Hopkins Medicine International Affiliate, Post Box 15258, Al Ain, UAE; 2 Al Daar Al Ain International School, Al Ain, UAE; 3 Department of Statistics, Gulf Medical University, Ajman, UAE; 4 Johns Hopkins Graduate School of Education, Baltimore, MD USA

**Keywords:** Health literacy, Middle Eastern, Survey instrument, Culture, Health outcomes

## Abstract

**Background:**

Health literacy is a term employed to assess the ability of people to meet the increasing demands related to health in a rapidly evolving society. Low health literacy can affect the social determinants of health, health outcomes and the use of healthcare services. The purpose of the study was to develop a survey construct to assess health literacy within the context of regional culture. Different socioeconomic status among the Eastern and Middle Eastern countries may restrict, health information access and utilization for those with low literacy.

**Methods:**

By employing expert panel, Delphi technique, focus group methodologies, and pre-testing using participants (*N* = 900) from the UAE and India, a survey construct to the Eastern-Middle Eastern cultures was developed. Reliability was assessed using Cronbach’s α and validity using Factor analysis. Kiaser-Meyer-Olkin (KMO) sampling adequacy and Bartlett’s tests were used to assess the strength of the relationship among the variables.

**Results:**

Inclusion of non-health related items were found to be critical in the authentic assessment of health literacy in the Eastern and Middle Eastern population given the influence of social desirability. Thirty-two percentage of the original 19-item construct was eliminated by the focus group for reasons of relevance and impact for the local culture. Field pretesting participants from two countries, indicated overall construct reliability (Cronbach’s α =0.85), validity and consistency (KMO value of 0.92 and Bartlett’s test of sphericity was significant).

**Conclusion:**

The Eastern-Middle Eastern Adult Health Literacy (EMAHL13), screening instrument is brief, simple, a useful indicator of whether or not a patient can read. It assessespatients’ ability to comprehend by distinguishing between health and non-health related items. The EMAHL13 will be a useful too for the reliable assessment of health literacy in countries, where culture plays a significant impact. This will be the first steptowards providing equitable access to healthcare for countries that have large populations with low socioeconomic status.

## Background

The rapid proliferation of healthcare information and unprecedented technological advances of the past few decades have greatly increased the complexity of healthcare delivery, requiring patients to play a more active role in the management of their health [1]. Many patients, particularly the elderly and the economically disadvantaged, face challenges in today’s healthcare environment because of this increasing sophistication of diagnostic and therapeutic procedures. Health literacy is a term employed to assess the ability of people to meet the increasing demands related to health in a rapidly evolving society [2]. Health literacy encompasses the cognitive skills that enable people to comprehend and use health information in order to preserve and promote good health [3, 4]. In recent years, health literacy has gained significant attention because of its close relationship with the social determinants of health, health outcomes and use of healthcare services [3, 4]. Low health literacy has been associated with poor health outcomes resulting from poor adherence to intake of medication, limited understanding of the health system, the inability to comprehend health-related instructions for self-management, especially in case of chronic diseases [1–5]. As an example the prevalence of daiabetes was high among the uneducated versus those with college degree in the US [5].

Globally, several instruments have been used to screen adult health literacy, though many differ in their purpose and design [6]. Despite the wide choice of available tools, mostof the standardized survey instruments were designed, developed and tested by and for English-speaking populations and may have limited utility in multi-ethnic, multicultural countries [7]. It is now recognized that many of the available health literacy tools do not capture all aspects of health literacy, and most fail to consider the impact of local setting or culture [8]. Without deference to cultural context, the assessment of health literacy inglobal populations, such as the Middle East, may be quite challenging [9]. With the priority of family over personal autonomy and the patriarchal and hierarchical social structure common in Eastern and Middle Eastern societies, health literacy screening may be also be influenced by social desirability, where survey participants provide socially acceptable responses [10].

In the 40 years since its independence, the United Arab Emirates (UAE), a Muslim nation with a mosaic of many ethnicities, languages and cultures, has created an infrastructure of healthcare services comparable with international standards [11]. The population of the UAE provides a distinct platform to understand health challenges and preferences related to the multi-ethnic populations of many Eastern and Middle Eastern cultures [11]. The UAE is home to over 80 nationalities. Its population is comprised primarily of Asians (46 %) and Middle Eastern Arabs (39 %), with UAE nationals representing less than 20 % of the total eight million residents [12]. Similar to many of its Gulf neighbors, thevast majority of the expatriate population in the UAE is made up of unskilled or semiskilled workers with limited formal education [11]. To date, there are no published studies of health literacy in the UAE. The purpose of the study was to develop a survey construct to assess low literacy within the context of regional culture, and in doing so, overcome the barriers of cultural differences and social desirabilty. Identifying health literacy levels will be a significant step towards providing equitable access to healthcare and health system utilization for both the affluent and the socio-economically challenged countries of the East and the Middle East.

## Method

The Eastern-Middle Eastern-Adult-Health Literacy 13 point Questionnaire (EMAHL13) was designed to effectively measure health literacy levels of patients in Eastern and Middle Eastern cultures. A three-stage methodology was used in developing this questionnaire. In the first stage, a literature review was performed and Expert panel discussions were conducted. In the second stage, the questionnaire was revised after focus group review. In the final stage, a pilot study (pretest) of the questionnaire was completed using participants (*N* = 900) from two countries, India and the UAE. The study was conducted at 2 Joint Commission International accredited academic medical centers in the emirate of Abu Dhabi, UAE. No personal identifiers were collected from the survey respondents to link them to their responses. Instead, participants were assigned a random number to record their response, coded by an independent coder and centrally archived at Tawam Hospital. The research protocol received ethics approved by the local Institutional Review Boards in both countries (AAMDHREC 13/55 & REC 01.10.2013RS279).

### Stage 1- expert panel

**Fig. 1:**
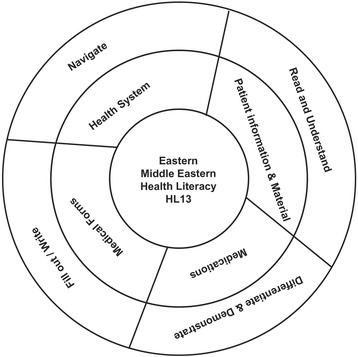
Conceptual framework for the Eastern-Middle Eastern adult health literacy (EMAHL13). Completing medical forms, reading patient information materials, navigating through the health care system and differentiating medications are the major activities through which patients engage with their health system and providers. Therefeore these were included as operational domains

An expert panel comprised of a purposive sample of 11 individuals, representing the fields of medical education (*n* = 2), public health (*n* = 3), and clinical research (*n* = 1) as well as local community members (*n* = 5), was convened to examine relevant domains in the assessment of health literacy in Eastern and Middle Eastern settings, with the explicit intent of minimizing the influence of social desirability by the survey participants in this cultural context. The experts were fluent in English and at least one other local language. Delphi technique, a group consensus gathering methodology, was used to reach agreement among the panel members [13]. After a comprehensive review of the literature pertaining to health literacy short screening tools, the content areas for inclusion in the questionnaire were primarily drawn from the Health Literacy Screening Brief Questions [14] to generate a conceptual framework relevant for Eastern and Middle Eastern cultures (Fig. 1).

### Stage 2- focus group

A focus group (*N* = 10), comprised of a convenience sample of students (*n* = 2), patients (*n* = 3), healthcare providers (*n* = 1) and members of the local community (*n* = 4), was conducted to provide feedback on the content and readability ease of the survey questions proposed by the expert panel [15]. Participants included both men and women between the ages of 18–50 years, who were able to understand, read and write English and at least 1 other local language. Participants especially from the community who were unable to consent were excluded. The Content Validity Index (CVI), a rating that the content is valid and consistent with the desired outcomes, was applied [16]. Accordingly, each member of the focus group was independently advised to score each item of the survey generated by the expert panel between 0–100 points based on relevance and importance to the local culture and setting, with higher scores representing greater relevance and importance of the survey item. Items obtaining a mean score of 80 % ormore were accepted as survey questions [16].

Since the populations of the Middle East and Asia are predominantly non-native English speaking, a readability test was then applied to all resulting survey questions. The Flesch-Kincaid scale was chosen because of its convenience for computerized use, excellent reproducibility and high correlation with other established readability scales [17]. The Flesch-Kincaid reading ease determines comprehension difficulty of written text on a scale of 0–100, with higher scores indicating that the material is easier to read. The Flesch-Kincaid grade level formula translates the score to a grade level, indicating the number of years of education generally required to understand a given text [17]. Question difficulty was included in the item development to ensure that the final items could distinguish between low, moderate and high levels of health literacy [18].

### Stage 3-pilot multicenter field Pre-testing

The revised version, containing the survey questions resulting from the focus group feedback, was used for the field pre-test. The pre-test was conducted using a purposeful sampling method, with the goal of including an equal distribution of participants with respect to age, gender and formal education. Patients from outpatient clinics from 2 major hospitals in the emirate of Abu Dhabi and 2 city hospitals in Mumbai, India were recruited for the study. Given the fact the study was conducted as the government-owned public hospitals in the UAE and India, a cross-section of urban and rural population participated in the study. Pilot field pre-testing involved face-to-face interviews between patient participants fromAbu Dhabi and Mumbai and the multilingual physician researchers. All respondents provided verbal consent prior to participation. A 5-point likert scale was used to assess participant responses [14, 18]. The response options included “*1 = never, 2 = rarely, 3 = sometimes, 4 = most of the time and 5 = always”* and the mean score for all item responses ranged from a minimumof 13 to a maximum of 65 for each participant*.* The scores distinguished between three levels of health literacy: 1–26 for “inadequate,”27-39 for “marginal” and 40–65 for “adequate” [19]. The researchers also used an electronic tablet device to display pictures of hospital signs, appointment slips, medical insurance forms, informed consent forms, English and local language newspaper reports, and local currencies for the purpose of identification and to assess comprehension.

### Translation

The final questionnaire EMAHL13 was translated from the original English version by certified health translators into the most prevalent languages in the UAE, namely Arabic, Hindi, Urdu, Tagalog and Malayalam. The surveys were then back-translated to English, in order to ensure that the structure, content and intent of the survey-items did not alter during translation. Although physician-researchers conducted the face-to-face interviews with the participants, the survey-items were translated into local languages to optimize participant comprehension.

### Data analysis

Data was analysed using SPSS Statistical Software Version 20 (SPSS Inc. Chicago, USA) [20]. The domains were identified using factor analysis [20]. The widely used Principal Component Analysis (PCA) was used as the extraction method to undertake factor analysis; and Varimax rotation was used to rotate the factors to better fit the data [20, 21]. Convergent validity to assess if the survey items converged to measure a construct was also conducted using the Correlation coefficient matrix method [20]. The percentage of total variance by each factor was calculated and pattern matrix was used to identify the domains. Kiaser-Meyer-Olkin (KMO) sampling adequacy and Bartlett’s tests (to assess the strength of the relationship among the variables) were also applied to the EMAHL13 [20, 21] . The reliability of the inventory and its subscales were tabulated using Cronbach’s alpha [20].

## Results

### Expert panel (Stage 1)

Characteristics of age, gender, nationalities, education and professional status of the members of the expert panel were equally distributed. Approximately half (6/11) of the expert panel members were women. Forty-five percent (5/11) of the panel was composed of community members, 27 % were public health professionals, and 9 % were healthcare providers. The average age of the participants was 37 ± 3.9 years, with 45 % having education levels at the high school level or below. The percentage of Asians and Arabs was almost equally distributed at 40 % each (data not shown).

The expert panel accepted 3 of the 5 functional domains from Chew et. al. Health Literacy Screening Brief Questions [14], specifically navigating the health care system, filling-out medical forms, and reading and understanding patient information materials. A fourth domain, differentiating and demonstrating medications and publication, consisted of non-health related items, such as the ability to read newspapers. Fifteen items were adapted from the 3 domains of the Health Literacy Screening Brief Questions, an additional 4 questions were derived from non-health related and medication domains, culminating in a 19-item culturally relevant health literacy survey (Table 1). Items from both the reading and understanding of patient materials domain (31.5 %) and medication instruction /non-health related domain (31.5 %) constituted the majority of the total items (Table 1). This was followed by 21 % of items from the filling-out medical forms domain. Only 16 % of the items were selected from the navigating through the health system domain (Table 1).

**Table 1:** Selection of the health literacy screening items relevant to the Eastern-Middle Eastern Cultures using the Focus Group (Stage 2)

Domain	Item#	Questions	CVI^a^ Score ± SD
Write/fill out	1	You can write your name and complete the treatment consent form	92.4 ± 8.7
	2	You can write and complete the past medical history form	93.5 ± 1.4
	3	You can write and complete the health insurance claim form for treatment	66.5 ± 3.0
	4	You can write and complete the patient registration form	64.1 ± 8.7
Differentiate & Demonstrate	5	You can understand and demonstrate reading an English newspaper	94.9 ± 1.9
	6	You can understand and differentiate the dosage instructions on the medication bottle label	95.3 ± 3.8
	7	You can understand and differentiate when and how medication needs to be taken from the prescription.	94.5 ± 2.2
	8	You can understand a pharmacy prescription	61.7 ± 3.5
	9	You can correctly demonstrate understanding of consumer product (detergent) information	70.7 ± 2.0
	10	You can understand and differentiate between the two similar medication labels	95.6 ± 2.4
Navigate the Health Care System	11	You can read and identify the hospital signs shown	95.9 ± 2.4
	12	You can read and dentify the out-patient clinic where you have your appointment	93.4 ± 2.4
	13	You can read and identify the medical supplies store	67.7 ± 3.7
Read & Understand	14	You can read and understand the appointment slip	96.4 ± 2.7
	15	You can read and understand the patient education material given to you	93.6 ± 2.8
	16	You can read and understand the Diagnosis instructions (full-bladder, overnight fasting) given to you	66.7 ± 3.4
	17	You can understand and demonstrate reading a local language newspaper	96.0 ± 2.5
	18	You can correctly demonstrate understanding of the local currency denominations given to you	96.5 ± 1.9
	19	You can read and understand patient rights and responsibilities sheet given to you	93.1 ± 2.5

^a^ The Content Validity Index (CVI) represents the average score rated by each member of the focus group for each item generated by the expert panel. The score between 0–100 points reflected the relevance and importance to the local culture and setting. Higher scores represented greater relevance and importance of the survey item. Items obtaining a mean score of 80 % or more were accepted as survey questions for field pretest

### Focus group

Sixty-percent of focus group participants were women, with an average age of 32.6 ± 3.8 (mean ± standard deviation). The average age of the male focus group participants was 37.8 ± 7.3. Sixty percent of the participants had a baccalaureate degree and were employed (data not shown). The focus group session provided feedback on the structure, content and clarity of the survey questionnaire. The questions were formulated as “you can” taking cues from the common local communication pattern in Eastern and Middle Eastern societies. The focus group participants reviewed the 19 semi-structured, open-ended items proposed by the expert panel and chose to include or exclude survey items based on their relevance and importance to the local population. Table 1 indicates the content validity index score for relevance and importance of the local setting. Survey items that obtained average scores less than 80 % were thereby removed from the questionnaire. Removed items included completing health insurance forms (66.5 %) and patient registration forms (64.1 %), reading pharmacy prescriptions (61.7 %) and medical supply store location (67.7 %), understanding consumer product information (70.7 %), and understanding diagnosis instructions (66.7 %). Structural revisions to avoid repetitive or duplication of the survey questions were also addressed by the focus group. The Flesch-Kincaid readability ease (FRES) testindicated a score of 61 and Flesch-Kincaid Grade Level (FGLS) of 7. It has been demonstrated that a reading score of 60 indicates standard readability ease [17].

### Multicenter field Pre-testing

**Fig. 2:**
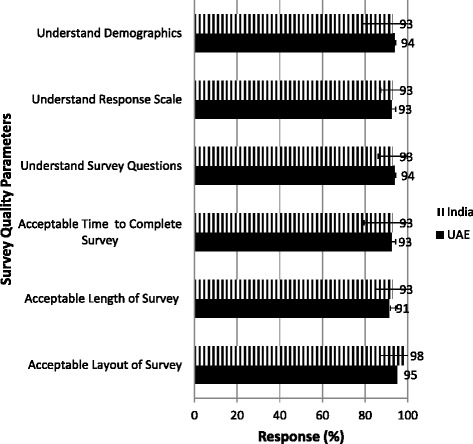
Quality assessment of the survey included survey length, completion time, understanding demographics, layout and scoring ease. There were no significant differences between the quality assessments of the survey instrument by the participants from either country. Responses are shown as average score ± standard deviation

The fieldtest participants were primarily women in both India (315/450, 70 %) and the UAE (272/450, 60 %). Compared to 66 % in the UAE, 80 % of the participants in India were employed. Quality assessment of the survey included survey length, completion time, understanding demographics, layout and scoring ease. There were no significant differences between the quality assessments of the survey instrument by the participants from either country (Fig. 2). The items related to the patient registration form, consumer product advertisement, medical store, pharmacy prescription, health claim form and diagnosis instructions were removed from the questionnaire in order to avoid duplication, as well as for their limited ability to directly assess health literacy (Table 2). The final health literacy construct (EMAHL13) is shown in Table 2. The average time required to complete the EMAHL13 survey was 11 min for participants from India and 13 min by UAE participants.

**Table 2:** The Eastern-Middle Eastern Adult Health Literacy (EMAHL13) Screening instrument for the population of the Eastern and Middle Eastern countries. The inclusion of non-health related items helps to unearth’s patient’s comprehension ability, possible reading impairments, and more importantly, to dissociate between education levels and health understanding and literacy

Domain	Item#	Questions	Responses				
Write/Fillout	1	You can write your name and Complete the treatment consent form	Never (1)	Rarely (2)	Sometimes (3)	Most times (4)	Always (5)
	2	You can write and complete the past medical history form	Never (1)	Rarely (2)	Sometimes (3)	Most times (4)	Always (5)
Differentiate & Demonstrate	3	You can understand and demonstrate reading the English Newspaper	Never (1)	Rarely (2)	Sometimes (3)	Most times (4)	Always (5)
	4	You can your understand and differentiate between the two similar medication labels	Never (1)	Rarely (2)	Sometimes (3)	Most times (4)	Always (5)
Navigate the Health Care System	5	You can read and identify the hospital signs shown	Never (1)	Rarely (2)	Sometimes (3)	Most times (4)	Always (5)
	6	You can read and Identify the out-patient clinic where you have your appointment	Never (1)	Rarely (2)	Sometimes (3)	Most times (4)	Always (5)
Read & Understand	7	You can understand and demonstrate reading the local language Newspaper	Never (1)	Rarely (2)	Sometimes (3)	Most times (4)	Always (5)
	8	You can correctly demonstrate understanding of the local currency denominations	Never (1)	Rarely (2)	Sometimes (3)	Most times (4)	Always (5)
	9	You can read and understand the appointment slip	Never (1)	Rarely (2)	Sometimes (3)	Most times (4)	Always (5)
	10	You can read and understand the patient education material given to you	Never (1)	Rarely (2)	Sometimes (3)	Most times (4)	Always (5)
	11	You can read and understand patient rights & responsibilities sheet	Never (1)	Rarely (2)	Sometimes (3)	Most times (4)	Always (5)
	12	You can understand and differentiate the dosage instructions on the medication bottle label	Never (1)	Rarely (2)	Sometimes (3)	Most times (4)	Always (5)
	13	You can understand and differentiate when and how medication needs to be taken from the prescription.	Never (1)	Rarely (2)	Sometimes (3)	Most times (4)	Always (5)

Validity and Reliability of the construct: The consistency of the health literacy scale (EMAHL13) was measured using the principal component analysis, obtaining a Kaiser-Meyer-Olkin value of 0.92, reaching statistical significance. Bartlett’s test of sphericity was significant (0.000). The principal component analysis revealed the presence of a component with an eigen value of 11.1, explaining 85.2 % of total variance, correlation coeeficient indicating relationship between the items are shown in Table 3. Cronbach’s α reliability assessment of each of the four domains ranged between 0.77 and 0.99 (Table 3) . The overall Cronbach’s α reliability score for all 13 items for the final construct was high at 0.85.

**Table 3:** Reliability and validity of the EMAHL13 construct. Internal consistency of the inventory and its subscales were tabulated using Cronbach’s alpha and, Principal Component Analysis was used as the extraction method to undertake factor analysis. The percentage of total variance by each factor was calculated and pattern matrix was used to identify the domains

Domain	Item#	Questions	Correlation Coeefcient	Cronbach’s Alpha
Write/Fillout	1	You can write your name and Complete the treatment consent form	0.87	0.78
	2	You can write and complete the past medical history form	0.79	
Differentiate & Demonstrate	3	You can understand and demonstrate reading the English Newspaper	0.97	0.97
	4	You can your understand and differentiate between the two similar medication labels	0.95	
Navigate the Health Care System	5	You can read and identify the hospital signs shown	0.96	0.99
	6	You can read and Identify the out-patient clinic where you have your appointment	0.96	
Read & Understand	7	You can understand and demonstrate reading the local language Newspaper	0.68	0.77
	8	You can correctly demonstrate understanding of the local currency denominations	0.78	
	9	You can read and understand the appointment slip	0.71	
	10	You can read and understand the patient education material given to you	0.62	
	11	You can read and understand patient rights & responsibilities sheet	0.74	
	12	You can understand and differentiate the dosage instructions on the medication bottle label	0.61	
	13	You can understand and differentiate when and how medication needs to be taken from the prescription.	0.5	

## Discussion

The scope and the number of surveys to measure health literacy have grown exponentially over the past decade [6]. However, survey designs in global cross-cultural environments have had limited success in the collection of high quality data [22], especially when comparison data are needed. Additionally, the current validated instruments to assess health literacy are either too long or too difficult to be routinely integrated into clinical care. Non-English speaking communities prefer to have survey questions that are brief, simply stated with easy layouts, and administered by local language speaking staff to enhance participant trust and confidence [22]. The EMAHL13 development has helped to overcome many of the above limitations.

Proper communication between patients and healthcare providers enables a shared understanding of patient values and treatment preferences that will lead to a care plan that is aligned with these values and preferences. Completing medical forms, reading patient information materials, navigating through the health care system and differentiating medications are the major activities through which patients engage with their health system and providers, for effective health services utilization [14]. The domains of the EMAHL13 was conceived with the objective of maximizing health services utilization and health promotion (Fig. 1).

The UAE provides an optimal venue to study opportunities and challenges related to multi-ethnic populations given the large number of non-nationals from the East and Middle East. The primary advantage of the EMAHL13 is that it is brief, takes only a few minutes to administer, a distinct advantage for assessing patient health literacy. The structure of the final questionnaire, which involves “you can” before every item, aided in limiting the influence of social desirability [23].

Obstacles to routine screening for health literacy include several factors, including patient attempts to conceal illiteracy, reading impairments, and difficulty in understanding the survey questionnaire [17, 24]. The inclusion of non-health related items, such as counting local currency and reading the local newspapers, helped to assess participants’ comprehension ability, possible reading impairments, and more importantly, helped to dissociate between education levels and health understanding, a significant step that will aid in health promotion.. Studies have indicated that a vast majority of patients in the United States read at grade 7 levels [17]. The readability ease (FRES) test indicated readability ease of 60 at grade level 7, signifying that the EMAHL13 would likely be easier for the local population to read. The 5-point “never to always” likert scale was used to provide distinct response choices for the participants in an effort to limit social desirability without affecting the survey reliability or criterion validity [7, 25]. The final construct met the reliability and validity criterion when compared with the Health Literacy Screening Brief Questions survey instrument. In both the UAE and India, the participants denied difficulty with the demographic questions, length and time to complete the survey, readability and comprehension, or layout and response choices.

Limitations of the study include the sampling methodologies, specifically convenience and purposive sampling, as the sample population may not be representative of all members of Eastern and Middle Eastern populations. However, these methods enabled an equal distribution of participants in regards to age, gender, ethnicities and education levels in the development of the survey construct. The EMAHL13, a researcher/interviewer-administered survey, also helped to overcome reliance on self-reported responses, which may be challenging in Eastern and Middle Eastern cultures. The blinding of the researcher/physician to the study hypothesis helped to overcome issues related to operator bias in administering the EMAHL13. The EMAHL13 is designed to be brief, simple, and serve not only as a useful indicator of whether or not a patient can read, but also provide information on the patient’s comprehension by distinguishing between health and non-health related items. Similar responses by the participants when the construct was tested in India and UAE indicate the flexibility, ease of understanding and feasibility of the instrument in the Eastern and Middle Eastern nations.

## Conclusion

As Eastern and Middle Eastern countries have vastly different socioeconomic situations, health information access and health system utilization may be particularly challenging for those with low health literacy. Priority of family over personal autonomy and the patriarchal and hierarchical social structurein the East further compounds the problem of health resources access and health utilization. It is anticipated that the EMAHL13 will prove to be a useful tool to measure limited health literacy in the Eastern and Middle Eastern populations. Assessing health literacy in the local population will be a significant step towards providing equitable access to healthcare and health services utilization for countries that have large populations with low socioeconomic status [26]. Health literacy screening will assist the Gulf Cooperation Countries, inclusive of the UAE, to efficiently manage resources for high health returns.
